# Overlapping enhancer/promoter and transcriptional termination signals in the lentiviral long terminal repeat

**DOI:** 10.1186/1742-4690-4-4

**Published:** 2007-01-22

**Authors:** Qing Yang, Aurore Lucas, Sodany Son, Lung-Ji Chang

**Affiliations:** 1 Department of Molecular Genetics and Microbiology, Powell Gene Therapy Center and McKnight Brain Institute, University of Florida, College of Medicine, Gainesville, Florida 32606, USA

## Abstract

Oncoretrovirus, but not lentivirus, displays a high transcriptional readthrough activity in the 3' long terminal repeat (LTR) (Zaiss et al. J. Virol. 76, 7209–7219, 2002). However, the U3-deleted, self-inactivating (SIN) lentiviral LTR also exhibits high transcriptional readthrough activity. Since the canonical "core" polyadenylation signal (AAUAAA) of the lentivirus is located in the R-U5 region, the above finding suggests that additional RNA termination signals must be present in the U3 region. Insertion of alternative termination signals including panhuman T cell leukemia virus type I polyadenylation signal, a 3' end small intron, and a tertiary tRNA motif into the lentiviral SIN LTR did not restore the transcriptional termination function. Functional dissection of the U3 region revealed that 70–80% of the termination signals reside in the transcriptional control region within 124 nt overlapping NFκB, Sp1 and TATA binding sites. Serial deletion analysis of the transcriptional control region indicates that the lentiviral enhancer/promoter elements are essential to the RNA termination function. These results characterize the mechanism of lentiviral transcriptional readthrough, which addresses important fundamental and practical issue of RNA readthrough influencing lentiviral gene function and vector safety.

## Findings

Lentiviral vectors (LVs) establish long-term transgene expression in both dividing and non-dividing cells. Extensive deletion of all of the viral genes and most of the LTR elements are essential to the safety of this vector system [1-3]. The self-inactivating vector (SIN) with minimal sequence in the viral LTR has been an important safety improvement in the LV system [4]. However, the LV SIN LTR displays very high transcriptional readthrough (TR) activity [5], which potentially increases the risk of activating downstream cellular oncogenes. Here we examined activities of potential transcriptional termination elements in the SIN LVs and functionally dissected U3 sequence to identify key transcriptional termination signals.

### Insertion of alternative transcriptional termination elements in the LV SIN LTR

**Figure 1:**
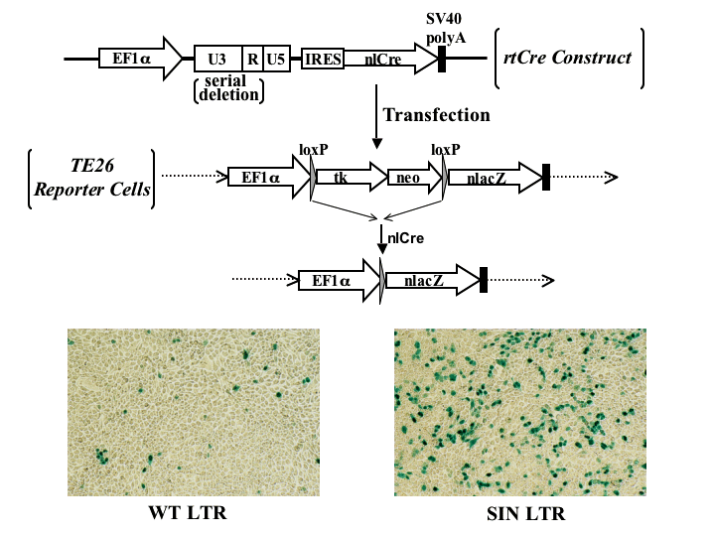
**The lentiviral LTR transcriptional readthrough assay**. The expression level of nlCre, which serves as an index of transcriptional readthrough of the LV LTR, is directly correlated with nlacZ expression in TE26 reporter cells as previously established [5]. The SIN LTR, which contains only the attL sequence of the U3, exhibits a high readthrough rate, while the WT LTR exhibits a low readthrough rate.

The RNA readthrough activity was determined using the sensitive Cre-loxP TE26 reporter cell line as previously described [5]. We transfected the readthrough reporter construct, EF-LTR-IRES-nlCre (rtCre), into TE26 cells which contain a loxP-nlacZ reporter gene whose expression closely correlates with the readthrough nlCre activity (Fig. 1). We confirmed that the WT LTR exhibits low transcriptional readthrough activity, whereas the SIN LTR exhibits high readthrough activity (Fig. 1, bottom).

**Figure 2:**
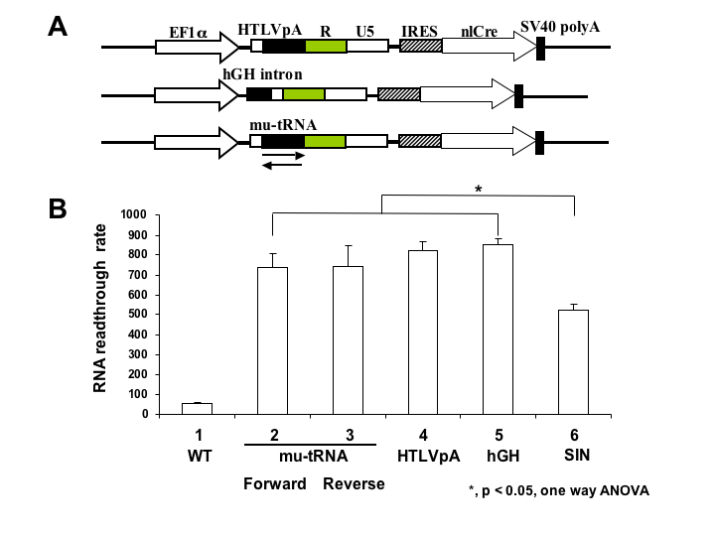
**Insertion of alternative termination signals fail to reduce the transcriptional readthrough activity of LV SIN LTR**. **A**. Schematic diagram of lentiviral SIN LTRs containing various termination signal elements. HTLVpA (a 130 bp fragment from MT4 cell genomic DNA) and hGH intron (a 193 bp fragment from pXO-hGH) were PCR amplified and cloned into pdl-EF-3'LTR-IRES-nlCre. The mutated tRNA (mu-tRNA, 236 nt) sequence was generated by annealing several synthetic oligos and cloned into LV SIN vector. All amplified fragments were verified by sequencing of the subclones and the final reporter clones. **B**. Quantitative analysis of termination signal insertion on SIN LTR readthrough. One-way ANOVA analysis reveals a significant difference between WT and the other groups (p < 0.05). No significant difference is detected between the different chimeric termination signal SIN LTR constructs. The modest difference seen between the chimeric termination signal SIN LTRs and the SIN LTR is significant (p < 0.05, marked by asterisk).

Transcriptional termination and RNA polyadenylation are regulated by different molecular mechanisms [6,7]. To reduce the readthrough rate of the LV SIN LTR, three different RNA termination signals were tested: the polyadenylation signal of HTLV-1 (HTLVpA), which is located in U3 rather than R-U5 of the HTLV-I LTR; the small intron near the 3' end of the human growth hormone gene (hGH intron), which may promote the RNA polymerase II termination function [8-12]; and a mutated tRNA motif (mu-tRNA), which forms a cloverleaf structure that may serve as a boundary element to block transcription (Fig. 2A) [11]. The nlCRE reporter constructs containing these individual elements (mu-tRNA, inserted in either forward or reverse orientation) were transfected into TE26 cells and the transcriptional readthrough activity was determined by nlacZ enzyme assay. When compared to WT and SIN LTRs, neither of these alternative termination signals restored transcriptional termination activity (Fig. 2B). Instead, all of these chimeric LTRs consistently exhibited increased readthrough activity (p < 0.05).

### Functional dissection of the lentiviral U3

**Figure 3:**
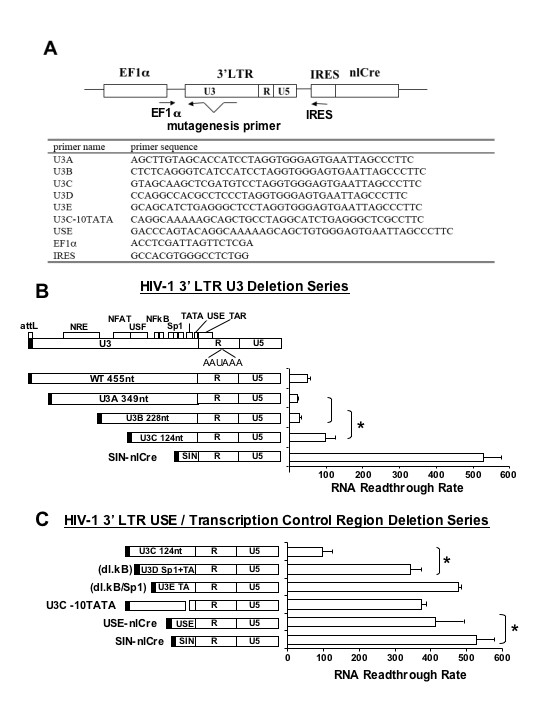
**Serial deletions in U3 and in the transcriptional control region and functional analysis of transcriptional readthrough**. **A**. The U3 deletion mutagenesis strategy and oligonucleotide primer list. The PCR strategy used for the generation of nlCre reporter constructs is illustrated. The first PCR product, generated by EF1α and a mutagenesis primer, was used as a mega-primer in a second PCR with the IRES primer to generate the DNA fragment for nlCre plasmid cloning. All mutagenesis constructs were verified by DNA sequencing. The amplified products were cloned into pdl-EF-3'LTR-IRES-nlCre (Fig. 1). **B. and C. **The 3' LTR deletion series. Genetic structure of the LTR U3 is illustrated at the top diagram. The deletion clones were generated in pBluescript subclones verified by squencing before being swapped into the final reporter construct. The reporter constructs were verified again by sequencing. TE26 cells were transfected with 0.1 or 0.2 μg of different plasmid DNA and 48 h later, fixed and stained with 5-bromo-4-chloro-3-indolyl-β-D-galactopyranoside, and the RNA readthrough rate was determined. The bar graphs to the right summarize the results of RNA readthrough analysis. The results shown are representative of more than three independent duplicate or triplicate transfections.

Several genetic elements in U3 and R regions of the human immunodeficiency virus type I (HIV-1) LTR have been shown to play a role in transcriptional termination [10][13-15]. To identify the U3 elements that are critical to transcriptional termination, we systemically restored WT U3 elements back into the SIN LTR in the nlCre reporter construct by sequential PCR (Fig. 3A) and tested their transcriptional readthrough activity (Fig. 3B &3C). The upstream signaling element (USE), located 77–94 nt 5' to the HIV-1 polyadenylation site (AAUAAA), has been shown to bind to the cleavage polyadenylation specificity factor (CPSF) and directly stabilizes the polymerase II polyadenylation complex formation [16]. USE has been reported to enhance HIV-1 3' RNA processing by approximately 70% [17,18]. We restored the full-length USE element and generated USE-nlCre (Fig. 3C bottom), and transfected TE26 cells with different amounts of USE-nlCre DNA and counted the blue-nucleated cells 48 h later. The results indicate that restoring USE reduced TR frequency by about 20% (Fig. 3C, USE-nlCre vs. SIN-nlCre, p < 0.05).

The transcriptional readthrough activity of USE-nlCRE was still 8-fold higher than that of the WT LTR. To identify additional termination signals, we generated a series of U3-restored rtCre reporter constructs (Fig. 3B). Different lengths of U3, 349 nt, 228 nt, and 124 nt, designated as U3A, U3B, and U3C, respectively, were inserted 5' to the R region in the SIN nlCre reporter construct as illustrated in Fig. 3B. U3C includes the two NFκB, three Sp1 binding sites, and the TATA Box. U3B extends further upstream to the NF-AT and USF binding sites. U3A retains most of U3 sequence including the 5' NRE. These constructs were transfected into TE26 cells and the TR activity was determined. The results reveal that the length of the U3 insertion is inversely correlated with the readthrough activity, suggesting that the termination signals spread across the entire U3. Nevertheless, the U3C construct exhibited a low RNA readthrough rate, 20 to 30% of the SIN-nlCre readthrough rate, close to that of the WT LTR (U3C 124 nt vs. SIN and WT 445 nt). Therefore, the majority activity of the termination signals appear to fall within the 124 nt of U3C.

### Analysis of key termination signals contributing to LV RNA readthrough

U3C contains NFκB and Sp1 binding sites and TATA box. To dissect the role of these transcription factor binding sites, we generated additional deletions in U3C, which included deletion of NFκB binding sites (U3D), deletion of NFκB and Sp1 binding sites (U3E), and deletion of TATA box alone (U3C-10 TATA), as shown in Fig. 3C. Results of transcriptional readthrough assays show that deletion of NFκB binding sites (U3D) significantly increases the RNA readthrough rate (p < 0.05), suggesting a critical role in transcriptional readthrough. Further deletion of Sp1 binding sites (U3E) follows the trend with more readthrough. Interestingly, the deletion of TATA box alone (U3C-10 TATA) with intact NFκB and Sp1 binding sites also results in high readthrough, indicating that the basic promoter element (or function) is critical to the transcriptional termination function. The deletion of all enhancer/promoter elements plus USE (SIN LTR) results in the highest RNA readthrough rate. Therefore, the HIV-1 enhancer/promoter elements are critical transcriptional termination elements.

### NFκB enhances the SIN LTR transcriptional termination function

**Figure 4:**
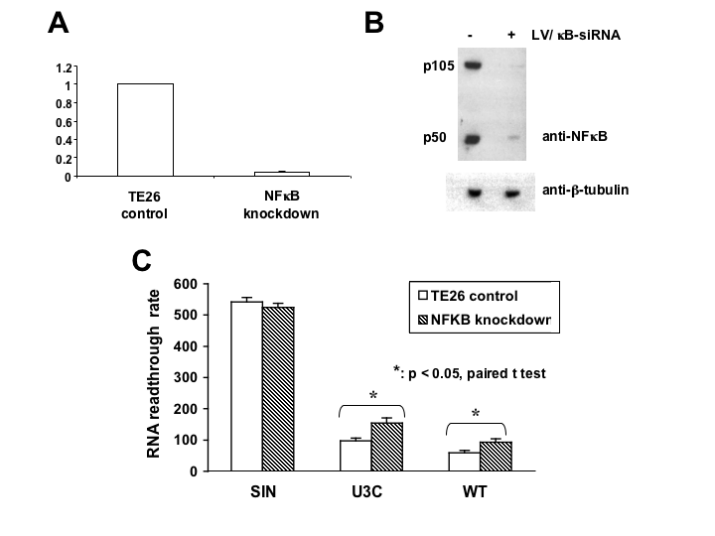
**Lentiviral siRNA silencing of NFκB reduces RNA readthrough of LV SIN LTR**. The NF kB siRNA lentiviral vector was made by annealing two oligos pre-treated at 95°C for 5 min and gradually cooled down to room temperature. The annealed products were cloned into pTYF-Puro-hU6r LV-driven by a human U6 promoter. The NFκB silenced TE26 cell line was generated after puromycin selection and used for RNA TR analysis as described in the article. **A. **Real-time quantitative RT-PCR analysis of NFκB mRNA. **B. **Western analysis of NFκB expression in LV-NFκB siRNA-transduced TE26 cells. **C. **Comparison of RNA readthrough rate in TE26 and NFκB-silenced TE26 cells. The significance of difference is analyzed by paired student t test and shown by asterisks.

The above U3 dissection study suggests that NFκB may play a critical role in the LV RNA 3' transcriptional termination function. To evaluate the relevance of the NFκB trans-acting factor, we silenced the expression of NFκB in TE26 using lentiviral siRNA targeting the p50 of the NFκB dimer and compared the readthrough efficiency of SIN LTR, WT LTR and U3C LTR in both control and NFκB-silenced TE26 cells. TE26 cells were transduced with lentiviral siRNA vector (LV/κB-siRNA with a puromycin-resistant gene) and after several passages under puromycin selection, the p50 NFκB RNA was quantified by real-time RT-PCR. The RNA analysis showed that the siRNA suppressed more than 95% of the NFκB RNA expression (Fig. 4A). This was confirmed by Western analysis using anti-NFκB antibody illustrating more than 95% inhibition of expression of both p105 and p50 of the NFkB family of proteins (Fig. 4B). To test the effect of NFκB on transcriptional readthrough, TE26 and TE26-siNFκB cells were transfected with different amount of SIN LTR, U3C LTR or WT LTR rtCre plasmids and the readthrough activities were determined. Repeated experiments demonstrate that in the NFκB-knockdown TE26 cells, the readthrough rate of U3C LTR and WT LTR, both of which contain two NFκB binding sites, is consistently up-regulated, but not for the SIN LTR which does not contain any NFκB binding site. Statistical analysis also supports that the readthrough rate in the NFκB-silenced TE26 cells is significantly increased for both the WT LTR (p < 0.05) and the U3C LTR (p < 0.1, paired t-test) constructs (Fig. 4C).

**Figure 5:**
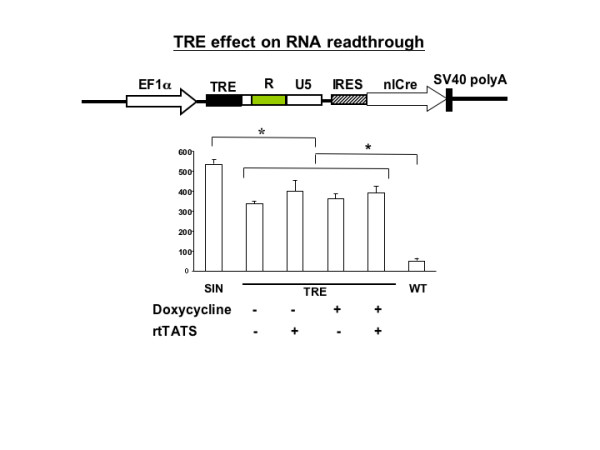
**Quantitative analysis of TR rate of LV SIN LTR containing TRE binding sites**. Tetracycline responsive elements (TRE) were derived from the *Xho *I to *Xma *I fragment of plasmid TREd2eGFP (BD Clontech) and ligated into the LV SIN vector. The RNA readthrough analysis was performed as previously described in the presence or absence of doxycycline as indicated. One-way ANOVA analysis shows a significant difference between the different TRE constructs and the SIN or WT LTR construct (p < 0.05).

The WT U3 contains multiple transcriptional factor binding sites. Our results suggest that the binding of transcriptional factors may contribute to the enhanced polymerase II termination function. To test if a foreign transcriptional factor binding site could block RNA readthrough, we inserted a synthetic seven-consecutive Tet-responsive element (TRE) in the SIN rtCre reporter plasmid and tested its readthrough activity in the presence or absence of a TRE-binding reverse tetracycline trans-activator/silencer (rtTATS). If the binding of rtTATS in the TRE region enhances transcriptional termination function, a reduced readthrough activity would be expected. The binding of rtTATS was induced by doxycycline. The result showed that insertion of TRE reduced the readthrough rate by around 20%, regardless of the presence of rtTATS (Fig. 5). In addition, induction of rtTATS binding to TRE did not influence the readthrough rate. Therefore, the binding of a non-native transcriptional factor in the SIN LTR did not significantly improve the RNA pol II termination activity.

Here we have illustrated that heterologous termination signal elements such as HTLVpA, a small 3' intron and a tertiary RNA motif (tRNA) do not restore LV RNA termination function. In addition to USE, two additional elements in U3, the transcriptional control region and the NFAT/USF binding region, contribute significantly to lentiviral LTR transcriptional termination (Fig. 3B). Restoration of transcriptional control region alone reduces readthrough by 70–80%, while insertion of NFAT/USF, an additional 100 nt upstream of the transcriptional control region, reduces RNA readthrough to a level even lower than that of the wild type LTR.

Further dissection of transcriptional control region indicates that the key enhancer/promoter elements overlap with the transcriptional termination elements, or that they have an overlapping function. The NFκB binding sites appear to have a dual role during HIV-1 transcription. It is possible that interaction of the NFkB elements directly or indirectly affect binding of the 3' RNA processing factors. In addition to the analysis of "cis-elements", the readthrough study with the NFκB siRNA supports that "trans-factors" also play a role. The artificial introduction of a transcriptional factor binding cassette (seven copies of the Tet-responsible element, TRE) into SIN LTR resulted in a modest decrease in RNA readthrough. These additional findings support that both "cis-elements" and "trans-elements" (transcriptional factors or DNA binding proteins) play an important role in lentiviral transcriptional readthrough.

The high RNA readthrough frequency of the lentiviral SIN vector could compromise the intended safety feature. This may be overcome by further characterization of the RNA transcription elongation and termination machinery and genetically modify the control elements to reduce readthrough without adverse effects. Recent studies have established that components of the pol II holoenzyme can interact with transcription factors, HIV-1 Tat as well as mRNA processing factors involving capping, splicing, termination and polyadenylation [19,20][21,22]. Further analysis of the transcriptional readthrough process of lentiviral LTR will address the fundamental mechanism of RNA termination and help design for a safer and more efficient lentiviral vector system.

## Abbreviations

HIV-1, human immunodeficiency virus type 1; LV, lentiviral vector; SIN, self-inactivating; WT, wild type; LTR, long terminal repeats; hGH, human growth hormone; HTLV, human T cell leukemia virus; TR, transcriptional readthrough; USE, upstream signal element; CPSF, cleavage polyadenylation specificity factor; TRE, tetracycline responsive element; ANOVA, analysis of variance.

## Competing interests

LJC has declared a financial interest as consultant to a company and LJC holds patents related to the work that is described in the present study.

## Authors' contributions

All authors participated in the molecular biology studies. LJC conceived the study. LJC, SS and AL initiated the design of the study and QY performed the statistical analysis. LJC and QY participated in the final figure preparation and drafted the manuscript. All authors read and approved the final manuscript.
